# Understanding the Political Organization and Tactics of the Alcohol Industry in Ireland 2009–2018

**DOI:** 10.15288/jsad.2022.83.574

**Published:** 2022-07-18

**Authors:** Matthew Lesch, Jim McCambridge

**Affiliations:** ^a^ Department of Health Sciences, University of York, York, United Kingdom.

## Abstract

**Objective:**

This study examines how the alcohol industry responded to developments in Irish alcohol policy leading to the 2018 Public Health (Alcohol) Act, a set of measures designed to reduce overall alcohol consumption in order to reduce harm to health and society. Previous research has emphasized the political and economic strengths of the alcohol industry in Ireland and elsewhere. This study examines the origins of and the debates over this legislation to better understand the political tactics of the alcohol industry.

**Method:**

The study focuses on developments between 2009 and 2018, tracing activities by industry actors to shape the policy process at different junctures. Data for the study are drawn from 18 semi-structured interviews with politicians, government advisors, public health experts, and advocates as well as from relevant primary documents, public statements, and newspaper articles.

**Results:**

The study identifies three interrelated tactics used by alcohol industry actors—obstruction through participation, coalition-building and mobilizing proxies, and making use of extensive political resources in lobbying—and traces their impacts at different points in the policy process. We find that industry actors had some success in influencing policy, defeating particular provisions by averting their inclusion, and winning amendments to others, but ultimately failed to defeat the legislation. Specific opportunities and constraints present in the Irish context for alcohol industry actors are identified.

**Conclusions:**

Public health considerations withstood a range of challenges from alcohol industry interests in passing public health legislation in Ireland. The findings have important implications for the study of the alcohol industry's political tactics in Ireland and elsewhere, including the use of lobbying registry data as a potential data source.

The alcohol industry has held considerable economic and political power in Ireland for centuries (Butler et al., 2017; Hope, 2006; Hope & Butler, 2010). Until recently, there had been no concerted effort to significantly reduce alcohol consumption in Ireland. By the late 2000s, however, concerns over rising alcohol consumption reached new heights and the Irish government looked for an alternative approach. Through efforts of the Department of Health, alcohol became increasingly defined as a threat to public health (Lesch & McCambridge, 2021a, 2021d, 2022). This reframing of alcohol contributed to a lengthy debate over whether Ireland should introduce new restrictions on the availability, price, and promotion of alcohol. Despite a highly organized opposition campaign led by the alcohol industry, the Public Health (Alcohol) Act was adopted in 2018. These recent developments provide an opportunity to better understand how the alcohol industry operates politically and to identify potential limits to its influence in the policy process.

A systematic review identifies two key strategies for the alcohol industry's influence on policymaking: (a) policy framing, in which industry actors shape ideas about alcohol, including problem-definition and policy alternatives; and (b) policy influencing, where industry actors use collective action or relationship building to advance their preferred policy outcomes (McCambridge et al., 2018). In the Irish context, industry actors have sought to keep policymakers’ attention on specific segments of the population, such as problem drinkers, and promote policy measures without strong evidence (e.g., education programs) (Calnan et al., 2018; Hope, 2006; Petticrew et al., 2016).

As in many producer countries, alcohol industry actors in Ireland have coordinated their political activities through various industry associations (McCambridge et al., 2018). The Drinks Industry Group of Ireland (DIGI) was formed to serve as the umbrella group for alcohol industry trade associations covering manufacturing, distribution, and on-premises/off-premises retail. Alcohol Beverage Federation of Ireland (ABFI) was a member of DIGI and was mainly concerned with representing the different sectors of production and distribution, and later rebranded in 2019 to Drinks Ireland. Industry actors also established a social aspects organization in 2002, the Mature Enjoyment of Alcohol in Society Limited. MEAS was responsible for introducing Ireland's Drinkaware website in 2006 and later changed its name to Drinkaware.ie. There are thus many apparent similarities between the extant work on Ireland and findings on the alcohol industry elsewhere (McCambridge et al., 2019; Mialon & McCambridge, 2018).

In 2009, as part of its National Substance Misuse Strategy (NSMS), the Irish government appointed a steering group to examine alcohol-related harms (Department of Health Ireland, 2012). The group comprised a range of policy actors from across Ireland, including representatives from several government departments, civil society organizations, and the alcohol industry. In its final report, released in 2012, the group made several alcohol policy recommendations, including the following: (a) minimum unit pricing (MUP), (b) the structural separation of alcohol from other products in retail, (c) new restrictions on alcohol advertising, (d) new labeling requirements on alcohol products, and (e) a ban on sports sponsorship by alcohol companies (Department of Health Ireland, 2012). Excluding sports sponsorship, these recommendations formed the basis of the 2018 legislation (Government of Ireland, 2018). Throughout the steering group's deliberations and the subsequent legislative debate, the alcohol industry served as a barrier to progress. Our analysis identifies the tactics used by alcohol industry actors to advance their interests.

The Irish case study presents an opportunity to better understand the alcohol industry's strategies and activities, including the opportunities and constraints these actors confront in the policy process (McCambridge et al., 2020). This analysis casts a wider net than previous research, exploring industry activities over an extended timeframe (although see Hawkins & McCambridge, 2020b), uniquely across a comprehensive range of policy measures. The article also uses several concepts from political science, including issue framing (Chong & Druckman, 2007), coalition building (Bernstein & Hoffmann, 2018; Hughes, 2019), and lobbying (De Figueiredo & Richter, 2014). This approach to analysis helps researchers to identify insights that are deep, nuanced, and also potentially generalizable to other policy contexts.

## Method

This article explores the alcohol industry's tactics in the context of the Public Health (Alcohol) Bill 2015. The analysis considers the period between 2009 and 2018, tracing the origins of the legislation and industry efforts to shape the policy process at different junctures (Beach & Pedersen, 2019). We draw on several data sources, including primary documents, secondary literature, and newspaper coverage. In addition, we undertake a thematic analysis of 18 semi-structured interviews conducted with key policy participants. Interviews were conducted between September 2019 and August 2020 in person or over Zoom. Interviewees were purposively sampled after having been identified through a combination of government documents, media reporting, and snowball sampling. The interviewees included public health advocates (*n* = 9), public health experts (*n* = 2), elected officials primarily from three political parties (Fine Gael, Labour, and Sinn Féin; *n* = 7), and political staff (*n* = 1). These individuals were recruited because they were either actively involved in the policymaking process or possessed in-depth knowledge of the developments in question. The Health Sciences Research Governance Committee at the University of York provided ethical approval for the study in February 2019.

Industry actors were not included as part of the interview sample. Instead, we explore how other policy participants perceived industry actors’ shorter term actions and decision-making, in the context of longer term strategies and tactics. This study uses non-industry interviewees’ perceptions of industry, along with other materials from and on industry actors. These other sources, including public statements made by industry actors and industry submissions to government, are used to triangulate findings from the interviews to ensure reliability.

The interviews were undertaken by the first author, recorded with permission, and transcribed verbatim. Transcripts were initially coded using NVivo 12 (QSR International, Melbourne, Australia) by the first author. A codebook was developed to organize and interpret the initial data, identifying key developments as they related to industry activities. The thematic content was subsequently refined and analyzed by both authors. Selected quotations from the interviews are included in a supplemental appendix that accompanies the article. (Supplemental material appears as an online-only addendum to this article on the journal's website.)

### Findings

*Obstruction through participation*. Between 2009 and 2012, the NSMS steering group met 20 times. According to one interviewee who served on this group, alcohol industry actors had used their seats at the table to stymie the process (Supplementary Appendix, Item A). Other interviewees described a collective sense of frustration with the ABFI and MEAS representatives, with one accusing them of “foot-dragging” (Interview-A). Another interviewee believed that the steering group's work could have “only required a few months of work” but that industry obstruction meant it took years to complete (Interview-B). Industry actors contested the terminology being used, for example, insisting that the term “alcohol misuse” be used in the group's terms of reference (Interview-C). When the steering group turned to specific research studies on alcohol policy alternatives, industry actors often presented findings that had either been taken out of context or were inconsistent with what had been identified in the scientific literature (Supplementary Appendix, Item B).

Eventually, however, the large majority of the group agreed to push forward after averting the industry's attempt to reframe key policy issues. Further attempts to delay the process were met with skepticism, indeed sometimes derision. As one interviewee explained: “when [industry representatives] kept asking for more evidence we eventually just said … ‘no, there is lots of evidence here … let's move it forward’” (Interview-A). In other instances, industry representatives presented their own research, inculcating doubts about the science (Supplementary Appendix, Item C).

Discussions on sports sponsorship had reportedly been particularly contentious. The alcohol industry found some sympathy among other participants in their opposition to a ban on sponsorship. Officials from the Departments of Transport; Tourism and Sport; and Arts, Heritage and the Gaeltacht (primarily Irish language–speaking areas) expressed concerns about the economic impacts of this policy. Officials eventually backed the majority's recommendations; but, as one participant explained, on this particular topic, “you would swear that they were working with the industry” (Interview-B).

After 20 meetings, the group summarized its key recommendations in a final report. The industry representatives, however, announced that they would decline to support it (Interview-C) and instead issued two minority reports to register their objections to the key recommendations (Alcohol Beverage Federation of Ireland, 2011; Mature Enjoyment of Alcohol in Society Limited, 2011). Even though there had been disagreements, this development offended several participants (Supplementary Appendix, Item D).

In October 2013, drawing primarily from the official report, the Coalition Government released its alcohol strategy proposals. The proposed plan would include four main policy changes: (a) MUP; (b) structural separation of alcohol from other products in shops; (c) advertising and marketing restrictions; and (d) health information on alcohol products and marketing, including health warnings and pregnancy advice. Although lacking the complete ban on sports sponsorship, the plan represented the first time alcohol would be specifically tackled as a “public health issue” in public policy (O’Keeffe, 2013).

Some of the policy proposals had a longer and more complicated history. The structural separation of alcohol, for example, had already been legislated as part of the Intoxicating Liquor Act 2008, yet the health minister at the time decided against commencing the provision (Butler, 2015). According to one interviewee, lobbying by the retailers had apparently contributed to this decision (Interview-B). Instead, the government and retailers agreed to a voluntary Code of Practice. To monitor the code's implementation and to provide regular reporting to the government, several retailers created a new organization, the Responsible Retailers of Alcohol in Ireland (RRAI) (Burns, 2009).

*Coalition-building and mobilizing proxies*. The ban on sports sponsorship was notably omitted from the government's legislative plan (McGee, 2013). Our findings suggest that the alcohol industry was instrumental in this decision. As former junior health minister, Róisín Shortall later recalled:

[D]epartmental officials enthusiastically got to work on drafting legislation to give effect to the proposals in the [NSMS] strategy. When I consulted members of the Cabinet, it … became … obvious that the alcohol industry had continued on its lobbying spree. It was particularly successful in targeting the … Ministers for Agriculture, Food and the Marine; Transport, Tourism and Sport; and Justice and Equality (House of the Oireachtas, 2018).

Following Shortall's resignation from the cabinet, alcohol policy continued to be debated. Shortall's successor, Alex White, emerged as the bill's key champion in cabinet. According to White, the alcohol industry and its allies continued to exert significant pressure on ministers (Supplementary Appendix, Item E).

During cabinet discussions, disagreement emerged between White and Leo Varadkar, the Transport, Tourism and Sport Minister. On one hand, two key national sporting organizations—the Irish Rugby Federation Union and the Football Association of Ireland—urged the government to drop the sports sponsorship ban (O’Keeffe, 2013). On the other hand, the Gaelic Athletic Association (2018) took a different position on the matter, refusing to accept alcohol industry sponsorship from 2014.

Interviewees described how effective the alcohol industry was in mobilizing sports groups with few resources that had historically benefited from industry sponsorship. These included the major national sporting associations who used their contacts and resources to lobby cabinet members. Moreover, local sporting clubs indirectly pressured the government by reaching out to elected representatives, in a culture of clientelism (Supplementary Appendix, Item F). One public health expert explained the political sensitivity of sports sponsorship: “industry [is] not advertising their wares, they’re giving money to sports … [and so] that's very hard to argue against” (Interview-B). Thus the alcohol industry was successful in mobilizing national and local-level organizations that had an interest in allowing sponsorship to continue. Another consequence of this success is that it made it challenging for public health advocates to garner the support of these sporting organizations to support other parts of the bill (Supplementary Appendix, Item G).

Experience with sports sponsorship contrasted somewhat with alcohol marketing restrictions. The new proposed restrictions on alcohol advertising included content regulations for alcohol advertising, a 9:00 p.m. watershed for alcohol advertising, and bans on advertising in specific locations, such as near playgrounds and at public transport stations. Unlike the sports sponsorship ban, these marketing restrictions, however, did not affect a broad range of stakeholders such as local businesses. On the issue of marketing, “there was no one else who would really be on [the industry's] side which … made it harder for them [to pressure the government]” (Interview-B). That being said, opposition to these provisions was not restricted to the alcohol industry. The marketing restrictions would have major financial implications for the advertising revenues of major media organizations, including RTE and TV3 as well Ireland's major newspapers (Power, 2017). Despite this, there is little indication that the alcohol industry and media organizations coordinated opposition to the bill's marketing provisions.

The alcohol industry's efforts to assemble different coalitions to oppose different parts of the alcohol plan continued during the legislative process. In October 2016, the alcohol bill was introduced in the Seanad Éireann (the upper house of the Irish legislature; O’Donovan, 2016). Structural separation became a key target for the alcohol industry. This required that alcohol be sold separately from other goods and entailed practical, as well as symbolic, change. The main retail trade associations claimed that the regulations would burden small businesses with new construction and labor costs (O’Donovan, 2016).

The alcohol industry seized on this issue by mobilizing small and rural alcohol retailers and encouraging them to pressure their elected representatives (Supplementary Appendix, Item H). This grassroots campaign was disclosed in the RRAI's submission to Ireland's Registry of Lobbying (Standards in Public Office Commission, 2017; Supplementary Appendix, Item I). One advocate recalled feeling “completely blindsided [by this] army of local shopkeepers” (Interview-C). Another interviewee described how the materials circulated by these local retailers often followed a similar “script and pattern” (Interview-I; Supplementary Appendix, Item J). Other interviewees stressed the dependence of Irish politicians on small business owners in rural areas for electoral support (Supplementary Appendix, Item K). One former political advisor in the Department of Health explained how this represented a common tactic, in which alcohol producers in Ireland turned to “surrogates” to help fight political battles (Supplementary Appendix, Item L). Others agreed with this interpretation, identifying the concentrated nature of the alcohol retail market in Ireland and its implications for political mobilization (Supplementary Appendix, Item M).

The structural separation issue stalled the legislation's progress in the Seanad. Several Fine Gael senators responded by threatening to vote against the government bill unless the provision was amended (Ryan, 2016). Senators tend to rely on political support from local councils, businesses, and voters, many of whom have direct or indirect links to the alcohol industry. Structural separation threatened to undermine the entire alcohol bill and served as a major political headache for the government. After several months, however, the health minister, Simon Harris, resolved the impasse, providing exceptions for smaller shops, primarily addressing the concerns of rural communities (McEnroe, 2017).

*Making use of the extensive political resources of industry in lobbying*. Alcohol industry actors also used direct lobbying as a way to influence the legislative process. The economic importance of the alcohol industry in Ireland, particularly large exporters such as Diageo, has long provided industry actors with “access” to key decision-makers (Interview-P). As one public health advocate explained: “The alcohol industry can knock on any door … in this country, and it’ll be opened for them” (Interview-E).

Some interviewees described how informal relationships between politicians and the industry enabled “soft approaches to lobbying,” meaning “access …. without necessarily declaring that access” (Interview-J). Some industry organizations also employed several former government advisors from the two major parties—Fine Gael or Fianna Fail. As one former Fine Gael advisor explained, the alcohol industry “literally hired [these advisors] one after another, which was all about access” (Interview-K). As described in the previous section, lobbying also occurred at the local level: “The alcohol industry [is] very savvy at that grassroots organising, so getting your little local shop to say, ‘if you have the minimum unit price, we will close down’” (Interview-F2). Although there is a reference to MUP in this quotation, it is striking how little this internationally salient issue was featured in Ireland. This was in some ways an issue that was challenging to industry unity; publicans were largely in favor of it, whereas the big producers were opposed.

Other interviewees pointed to parliamentarians’ incentives to be responsive to industry arguments: “The industry did a very good job of illustrating to politicians exactly how much they contributed to each economy and each constituency” (Interview-L and Supplementary Appendix, Item N). This was significant because no major party opposed the legislation. In fact, an informal all-party group of senators and Teachtaí Dálas (members of the lower house of the Irish Parliament) formed in 2013, with the sole purpose of advancing the bill. These circumstances meant that influencing individual parliamentarians offered the only available channel for the opposition (Supplementary Appendix, Item O).

ABFI also mobilized resources to pressure ministers or other high-ranking officials in a multilevel approach. As one politician recalled: “You could hear the arguments of the industry coming through when stuff was said to you by [other] officials” (Interview-D).

In 2015, a lobbying registry took effect in Ireland, requiring all lobbying activities to be publicly disclosed. This legislation was largely instigated by the Labour Party in the Coalition Government, which had also been the key early champions of the alcohol bill. Information from the registry was then made publicly available at the end of every quarter. The alcohol industry's lobbying efforts attracted media coverage (McGee, 2017; O’Halloran, 2018b; *The Irish Times*, 2018). According to one investigation, the Irish Business and Employers Confederation (IBEC), the parent organization of ABFI, had more than 100 entries into the registry, including several individual face-to-face meetings with the health minister, finance minister, and Taoiseach (Prime Minister). The investigation also showed that the Taoiseach, Enda Kenny, had met with the Chief Executive of Pernod Ricard (Cullen, 2016), a major global spirits producer. During the legislative debate, proponents of the bill pointed to these stories and other evidence of lobbying “in the form of face-to-face meetings, receptions, letters, emails, contact through social media” (House of the Oireachtas, 2018).

One public health advocate explained how the new lobbying legislation shifted the political dynamic in favor of the legislation's proponents. The requirement to disclose all communications meant that politicians did not want to risk appearing to be too close to industry:

We had the Lobbying Act come in around this time … so for the first time … there was a degree of transparency with the lobbying that went on … the lobbying register was important because politicians changed their behavior … they didn't want to be seen to be consulting with [industry] in the way they were before (Interview-M).

Lobbying was not always carried out directly by alcohol producers or industry associations. As the legislation progressed, professional lobbyists or public relations firms were increasingly relied on (Interview-O). The need to persuade several parliamentarians rather than individual decision-makers entailed a major effort (Supplementary Appendix, Item P). IBEC/ABFI was the key vehicle for industry lobbying, but they were far from alone (Supplementary Appendix, Item Q).

These lobbying efforts were associated with changes to the content of the legislation in various ways. The RRAI, for example, advocated for several changes to the legislation, many of which were subsequently proposed by senators as amendments to the bill. One lawmaker described these industry-friendly amendments as a “cut-and-paste job” (House of the Oireachtas, 2018).

Industry actors were ultimately unsuccessful in preventing the alcohol legislation from being passed by both houses of parliament (McGee, 2018). After nearly 3 years of debate in the legislature, the bill was enacted into law in October 2018 (O’Halloran, 2018a). Since then, the Department of Health has implemented different parts of the legislation on a staggered basis, with advertising restrictions taking effect in 2019, structural separation commencing in 2020, and MUP starting in 2022. Other provisions, such as product labeling, have yet to commence.

## Discussion

This study investigates how alcohol industry actors sought to advance their interests within the context of a debate over a historic piece of public health legislation. Our findings reveal three interrelated tactics used by the alcohol industry: obstruction through participation, coalition building, and well-resourced lobbying. Consistent with earlier work on this topic, the interviews portrayed the industry as formidable, highly strategic, and committed to using their resources extensively in the policy process. The strength of the industry, as indicated by the magnitude of its lobbying operations, was validated across several data sources.

The alcohol industry exhibited some success in influencing the policy process, but also clearly failed to defeat the bill. Ease of access facilitated a flexible and seemingly piecemeal approach to strategy, targeting different provisions of the bill at different stages in the process, and was necessitated by the unusual conditions operating in Ireland. Specifically, there was no divide between the major parties on alcohol; thus, individual parliamentarians had to be targeted. Additionally, the fact that the legislation included several distinct provisions meant that there were a series of battles to be fought. Each of these provisions had varying implications for different sections of the industry. This meant that different coalitions were built and different actors mobilized, as the legislation progressed. Given the cultural significance of sport, it is noteworthy both that this was the first major battle and also that it was won by industry. The public health interviewees largely saw themselves as being at war with the industry, and it may be that this was a long-term consequence of the obstructive tactics used during the Steering Group process.

The findings also strongly indicate that industry tactics should be conceptualized as interrelated and operating on at least two levels. One of the most important activities was in mobilizing local opposition, specifically local sports clubs in the case of sports sponsorship and then local retailers during the structural separation debate. This illustrates how different individual strategic elements—collective action, framing, and lobbying—can be pursued in conjunction with each other in search of synergies. Mobilizing these constituencies helped the industry to shift the framing of the debate away from health harms and toward economic impacts. Furthermore, stressing the local impacts of the legislation also provided the industry with leverage in their lobbying of politicians. This could be regarded as a multilevel strategy that is developed to align with the specific nature and incentives of the Irish political system. Yet this finding might also be generalizable to other national contexts, perhaps most strongly where there are similarities in local/national configurations. Future studies should consider how different industry political tactics can be designed to work in unison, as in this case, with what coherence and with what success. Such a study also needs to take account of how unity is forged and maintained, or not when the interests of different sections of the alcohol industry come into conflict with each other (Holden et al., 2012). This was the case here for MUP, and the potential for intra-industry conflict may be greatest in comprehensive alcohol policies, as was the case here.

These findings strengthen understanding of the alcohol industry's use of “proxies” in the battle for public opinion (Savell et al., 2016). Industry actors consistently used groups who were more likely to garner public sympathy. Existing work on the alcohol industry has identified collective action as a key industry tactic. Our findings demonstrate that the type of constituency that industry can mobilize, and with what level of success, has important political implications.

Adams (2016) examines issues associated with conflict of interest and identifies that industry funding can not only adversely affect an organization's reputation but also threaten financial independence. Here, specifically for local sporting clubs, there is an additional implication: Accepting industry funding induced expectations of reciprocity, in which support for policy positions was expected.

Our analysis also provides insights into how alcohol industry actors acquire expertise about the political system, employing previous government advisors for the purpose of lobbying. A related article has identified a similar strategy being used by the public health advocacy coalition (Lesch & McCambridge, 2021a). This suggests that both coalitions turned to actors who had intimate knowledge of the inner workings of the political system and enjoyed existing relationships with government officials. This might reflect Ireland's small size and the relatively small number of individuals who work in politics and public affairs to some extent, although this phenomenon is not restricted to such small countries (McCambridge et al., 2014).

This study identifies specific constraints facing the alcohol industry in its efforts to shape policy. Previous research has emphasized the strength of the alcohol industry in Ireland (Butler, 2009, 2015; Butler et al., 2017; Hope & Butler, 2010; Mercille, 2016). We show how this influence can be mitigated as political conditions change. First, the industry faced political leadership within the government that was determined to see the bill through. Since Fine Gael is a right-of-center party, there was limited electoral risk in being framed as anti-industry, as has been the case in other contexts. Second, the accumulated evidence on alcohol harm and the need to act to reduce it was foundational to the general public's support for the government's action (Lesch & McCambridge, 2021a, 2021d). The alcohol bill promised to address, even if not entirely resolve, the perennial challenges posed by a history and culture of heavy drinking in Ireland. Thus, despite their best efforts to delay or derail the legislation, industry confronted a political climate that was antithetical to its interests, and indeed this was too strong for it to prevail.

One potential limitation of the study is that industry actors were not included as part of the interview sample. Despite this, the analysis draws on a range of data sources to generate inferences about industry activity. Several key actors who engaged with industry actors and/or observed their behavior over an extended period were included in the study. Moreover, the study examined public statements made by alcohol industry actors in newspapers, reports, and press releases. Finally, the benefits of including industry actors in the interview sample need to be considered alongside potential risks, including respondent reliability.

The findings presented have important implications for the future study of the alcohol industry and its influence on policy, and also for arresting such influence. First, the introduction of disclosure laws, including lobby registries, was clearly important here. They have increasingly been used as a data source for studying interest group influence on policy (De Figueiredo & Richter, 2014) and are beginning to be used for the alcohol industry (Kypri et al., 2019). This article suggests that lobbying registries could also serve as potential objects of analysis within a particular case study. Here the introduction of the registry was a major asset for public health advocates. By making the alcohol industry's lobbying activities more visible, the registry shifted power dynamics in the policymaking context. The introduction of the registry may have made individual parliamentarians in Ireland more cautious in dealing with industry lobbyists. This raises important questions about how transparency and salience affect the responsiveness of politicians to the alcohol industry or other interest group pressure. Future work could consider how the presence of disclosure laws in other contexts has influenced the political power of the alcohol industry.

Finally, existing alcohol policy research has often focused on cases where a single policy instrument, such as MUP in Scotland or Wales, is contested by industry (Hawkins & McCambridge, 2020b; Lesch & McCambridge, 2021b). The Ireland case is potentially instructive in that it reveals how more comprehensive policy proposals generate different patterns of politics. In our analysis, the alcohol industry was successful in lobbying against the ban on sports sponsorship; this was accomplished through a combination of framing, coalition building, and high-level lobbying. In the case of structural separation, the legislation was framed as an attack on rural retailers, forcing the government to amend the provision, and thus providing evidence of some impact of industry tactics. In contrast, the advertising industry was not effectively mobilized to avert the introduction of a watershed time for alcohol advertising on traditional media.

These findings may be generalizable to other contexts. They suggest that the strength of industry influence on policy may be dependent on which parts of the alcohol industry, and which closely related sectors, are implicated, and mobilized by the threats posed by policy change. Is it also notable that the industry chose not to contest MUP in Ireland. In other contexts, particularly Scotland and England, the industry has focused its efforts on defeating MUP (Hawkins & Mc-Cambridge, 2020a; Katikireddi et al., 2014b), although the long-running contemporaneous legal case in Scotland may at least to some extent explain the weakness of opposition in Ireland and also in Wales (Lesch & McCambridge, 2021c). Future research should consider the reality that domestic alcohol policy unfolds within a broader regional and global policy context. The alcohol industry seems highly attuned to these dynamics, and alcohol policy researchers should follow their example.
